# Internal validation and comparison of the prognostic performance of models based on six emergency scoring systems to predict in-hospital mortality in the emergency department

**DOI:** 10.1186/s12873-021-00459-7

**Published:** 2021-06-10

**Authors:** Zahra Rahmatinejad, Fariba Tohidinezhad, Fatemeh Rahmatinejad, Saeid Eslami, Ali Pourmand, Ameen Abu-Hanna, Hamidreza Reihani

**Affiliations:** 1 Department of Medical Informatics, Faculty of Medicine, Mashhad University of Medical Sciences, Mashhad, Iran; 2 Department of Health Information Technology, Faculty of Paramedical Sciences, Mashhad University of Medical Sciences, Mashhad, Iran; 3 Department of Medical Informatics, Academic Medical Center, University of Amsterdam, Meibergdreef 9, Amsterdam, AZ 1105 the Netherlands; 4 Pharmaceutical Research Center, Pharmaceutical Research Institute, Mashhad University of Medical Sciences, Mashhad, Iran; 5 Department of Emergency Medicine, The George Washington University, School of Medicine and Health Sciences, Washington DC, USA; 6 Department of Emergency Medicine, Faculty of Medicine, Mashhad University of Medical Sciences, Mashhad, Iran

**Keywords:** Emergency department, Prognostic models, Performance measures

## Abstract

**Background:**

Medical scoring systems are potentially useful to make optimal use of available resources. A variety of models have been developed for illness measurement and stratification of patients in Emergency Departments (EDs). This study was aimed to compare the predictive performance of the following six scoring systems: Simple Clinical Score (SCS), Worthing physiological Score (WPS), Rapid Acute Physiology Score (RAPS), Rapid Emergency Medicine Score (REMS), Modified Early Warning Score (MEWS), and Routine Laboratory Data (RLD) to predict in-hospital mortality.

**Methods:**

A prospective single-center observational study was conducted from March 2016 to March 2017 in Edalatian ED in Emam Reza Hospital, located in the northeast of Iran. All variables needed to calculate the models were recorded at the time of admission and logistic regression was used to develop the models’ prediction probabilities. The Area Under the Curve for Receiver Operating Characteristic (AUC-ROC) and Precision-Recall curves (AUC-PR), Brier Score (BS), and calibration plots were used to assess the models’ performance. Internal validation was obtained by 1000 bootstrap samples. Pairwise comparison of AUC-ROC was based on the DeLong test.

**Results:**

A total of 2205 patients participated in this study with a mean age of 61.8 ± 18.5 years. About 19% of the patients died in the hospital. Approximately 53% of the participants were male. The discrimination ability of SCS, WPS, RAPS, REMS, MEWS, and RLD methods were 0.714, 0.727, 0.661, 0.678, 0.698, and 0.656, respectively. Additionally, the AUC-PR of SCS, WPS, RAPS, REMS, EWS, and RLD were 0.39, 0.42, 0.35, 0.34, 0.36, and 0.33 respectively. Moreover, BS was 0.1459 for SCS, 0.1713 for WPS, 0.0908 for RAPS, 0.1044 for REMS, 0.1158 for MEWS, and 0.073 for RLD. Results of pairwise comparison which was performed for all models revealed that there was no significant difference between the SCS and WPS. The calibration plots demonstrated a relatively good concordance between the actual and predicted probability of non-survival for the SCS and WPS models.

**Conclusion:**

Both SCS and WPS demonstrated fair discrimination and good calibration, which were superior to the other models. Further recalibration is however still required to improve the predictive performance of all available models and their use in clinical practice is still unwarranted.

## Introduction

Emergency Departments (EDs) are considered as frontline in clinical practice to provide critical medical care [1]. A number of models have been developed to classify patients with different acuity levels. Accurate and reliable models with minimum prediction error will help clinicians to prioritize patients correctly [2]. Scoring systems are potentially useful to provide relevant information on the severity of diseases, prioritize patients, determine the prognosis of patients, evaluate the quality of care, and optimize resource allocation [3–5]. There is also evidence showing that in a critical care setting, where physicians assess patients at high risk of deterioration, applying scoring systems is a useful mean along with clinical experience to facilitate distinguishing high-risk patients from low-risk ones [6].

Systems such as Acute Physiology and Chronic Health Evaluation (APACHE) [7], Simplified Acute Physiology Score (SAPS) [8], and Sequential Organ Failure Assessment (SOFA) were first introduced in the intensive care unit (ICU) [9]. Later, several scoring systems have emerged in the emergency department (ED) to risk stratify patients and predict mortality. These later systems includr the Simple Clinical Score (SCS) [10] Worthing Physiological Scoring system (WPS) [11], Rapid Acute Physiology Score (RAPS) [12], Rapid Emergency Medicine Score (REMS) [13], Modified Early Warning Score (MEWS) [14], and Routine Laboratory Data (RLD) [15], which have also been validated as the Biochemistry and Hematology Outcome Model (BHOM) [16]. Table 1 displays these models in terms of their variables and their point assignment scheme. As key variables they primarily include vital signs. Some subjective variables are also used by the SCS, such as ‘abnormal EKG’, ‘Unable to stand unaided or nursing home resident’, ‘underlying diseases’, and ‘spent some part of daytime in bed’ [3]. The RLD, in contrast, mainly includes laboratory parameters. However, The application of these models for outcome estimation on populations presenting to the ED has received much less consideration. Most of the previous studies were focused on just one specific group of disease or considered one or two types of these models. In this paper, we therefore design and perform a study to inspect and compare the performance of the six ED scoring models (SCS, WPS, RAPS, REMS, MEWS, and RLD) to predict in-hospital mortality using a large cohort of patients presented to a general ED.

**Table 1:** The point assignment scheme of each scoring system

Model (Min-Max)	Variables								Age (year)
	Temp(^0^C)	SBP (mm Hg)	MAP (mm Hg)	RR (breaths/min)	Pulse (beats/min)	GCS	AVPU	O_**2**_ sat(%)	
**SCS**	<35 or ≥39→2	>100→0	NA	<20 0	Pulse>SBP→2	Coma without overdose/intoxication→4	NA	≥95→ 0	male <50 or female <55 →0
		>80 & ≤100→2		>20 & ≤30→1					
				>30→2		Altered mental status without coma, overdose/ intoxication & aged ≥50→2		≥90→1	male ≥ 50 or female≥55 →2
		≥70 & ≤80→3		Breathless →1				<95→1	
								<90 →2	both male & female >75→ 4
		<70→4							
	Other items: New stroke on presentation→3 Unable to stand unaided, or a nursing home resident→2 Prior to current illness, spent some part of daytime in bed→2								
**WPS (0-14)**	≥35.3→0	≥100→0	NA	≤19→0	≤101→0	NA	A→0	96-100→0	NA
	<35.3→3	≤99→2		20-21→1	≥102→1		Other→3		
				≥22→2				94-95→1	
								92-93→2	
								<92→3	
**RAPS (0-16)**	NA	NA	70-109→0	12-24→0	70-109→0	≥14→0	NA	NA	NA
			50-69→2	10-11→1	50-69→2	11-13→1			
			110-129→2	25-34→1	110-139→2	8-10→2			
			130-159→3	6-9→2	40-54→3	5-7→3			
			≤49→4	35-49→3	140-179→3	≤4→4			
			≥160→4	≥5→4	≤39→4				
				≤50→4	≥180→4				
**REMS (0-26)**	NA	NA	70-109→0	12-24→0	70-109→0	≥14→0	NA	>89→0	<45→0
			50-69→2	10-11→1	50-69→2	11-13→1		86-89→1	45-54→2
			110-129→2	25-34→1	110-139→2	8-10→2		75-85→3	55-64→3
			130-159→3	6-9→2	40-54→3	5-7→3		<75→4	65-73→5
			≤49→4	35-49→3	140-179→3	≤4→4			≥74→6
			≥160→4	≥5→4	≤39→4				
				≤50→4	≥180→4				
**MEWS (0-19)**	≤35→2	≤70→3	NA	<9→2	<40→2	NA	Alert→0	NA	NA
	35-38.4→0	71-80→2		9-14→0	41-50→1		React to voice→1		
	≥38.5→2	81-100→1		15-20→1	51-100→0		React to pain→2		
		11-109→0		21-29→2	101-110→1		unresponsive→3		
		≥200→3		≥30→3	111-129→2				
					≥130→3				
**RLD**	−10.192 + (−0.013 × gender) + (5.712 × mode of admission) + (0.053 × age on admission) +(0.018 × urea) +(−0.001 × Na+) + (−0.101 × K+) + (−0.047 × albumin) + (−0.037 ×hemoglobin) + (0.067 × white cell count) + (0.001 × creatinine) + (2.744 × urea/creatinine)								

*Abbreviations*: *SCS* simple Clinical Score, *WPS* Worthing Physiological Scoring system, *RAPS* Rapid Acute Physiology Score, *REMS* Rapid Emergency Medicine Score, *MEWS* Modified Early Warning Score, *RLD* Routine Laboratory Data, *Temp* temperature, *SBP* systolic blood pressure, *MAP* mean arterial pressure, *RR* respiratory rate, *GCS* Glasco coma score, *AVPU* alert, voice, pain, unresponsive, *O2sat* oxygen saturation

## Methods

### Study design and settings

This prospective cohort study was performed from March 2016 to March 2017 in the Edalatian ED located in Emam Reza referral university hospital in Mashhad, northeast of Iran. The study was approved by the institutional review board of Mashhad University of Medical Sciences (ID:990106, IR.MUMS.fm.REC.1395.16) and conformed to the Declaration of Helsinki principles. The need for informed consent was waived by the Ethics Committee of Mashhad University of Medical Sciences because of the nature of the study and the analysis used anonymous clinical data.

### Inclusion and exclusion criteria

All adult patients (18 years of age or older) with high triage levels (Emergency Severity Index, ESI 1 to 3) were included in this study. The Patients who were discharged within 4 h after admission, readmitted with the same diagnosis, or died upon arrival were excluded. Moreover, patients requiring immediate surgical interventions (e.g. appendectomy), patients admitted due to traumatic or poisoning events, and patients with obstetric or ENT (Ear, Nose, or Throat) disorders were referred to their special wards and were consequently excluded from the study. The information about inclusion and exclusion criteria was reported previously [17].

### Study variables

The following variables were recorded at the time of admission: age, gender, vital signs (i.e., systolic and diastolic blood pressure, pulse rate, respiratory rate, temperature, AVPU, GCS score), mechanical ventilation status, oxygen saturation, abnormal electrocardiography findings (diagnosis made by the emergency medicine specialist), history of underlying diseases such as disability to stand without physical support, diabetes, new stroke or current apnea. The study end-point was in-hospital mortality. Moreover, the following laboratory results were measured using the serum which was obtained at the time of admission: serum urea, creatinine, sodium, potassium, albumin, white blood cell, hemoglobin, and platelet. All Collected variables were used to calculate the SCS, WPS, RAPS, REMS, MEWS, and RLD scores.

### Statistical analysis

Descriptive statistics were used to summarize characteristics of the study sample (i.e. continuous variables were expressed as Mean ± SD and categorical variables were reported in frequencies and percentages).

Logistic regression was used to develop models including each of the scoring systems. The predicted probability for each particular patient was calculated using the following formula:

$$ \mathrm{P}=\frac{1}{1+\exp \left[-\left({\beta}_0+{\beta}_1{X}_1\right)\right]} $$

(β_0_: Intercept; β_1_: Coefficient of the score; X_1_: score)

Each model was assessed in terms of discrimination, balance between sensitivity and positive prediction value, calibration, and accuracy of the predictions. Discrimination was measured by the area under the receiver operating characteristic curve (AUC-ROC). It is a performance measure which represents the ability of the model to assign higher probability of mortality for those who died than those who survived. The greater the AUC-ROC, the better the model’s performance at distinguishing between survival and non-survival cases. Balance between sensitivity (“recall”) and the positive prediction value (“precision”) was inspected by the Precision-Recall (PR) curve and measured by its corresponding area under the PRC (AUPRC). The lower the PPV the higher the recall. Knowing when the PPV begins to drop sharply may help one to select a suitable threshold on the predicted probability.

Calibration was assessed by calibration graphs (calibration refers to the *agreement between the predicted mortality and the observed* and mortality (as *estimated by the proportion of deceased patients*). For example, if one expects a 24% chance of mortality for a sub-group of patients, the observed mortality rate should be about 24 out of 100 patients. Calibration can be visually measured, in a plot with predictions on the x-axis and the proportion of outcome on the y-axis. An ideal calibration implies points on the diagonal (45°) line.

We used 1000 bootstrap replicates to generate smooth calibration plots that represent the degree of agreement between the observed and predicted probabilities. Points on the 45° diagonal line show perfect agreement. The Brier Score (BS) was also measured which is a measure of the accuracy of the predicted probabilities. It is the mean quadratic difference between the predicted probability and the respective observed outcome. The lower the Brier score, the better.

Internal validation of the performance measures was achieved by 1000 bootstrap samples. In each sample a logistic regression model was fit and its performance on both the bootstrap sample itself and on the original dataset was calculated. The mean difference between these two estimates over all bootstrap samples is a measure of optimism. This optimism is subtracted from the apparent performance of the final model that is developed and tested on the original dataset. We report the final model along with its optimism-corrected performance along with its 95% confidence interval. This interval is based on the percentile method in which the highest and lowest 2.5% of the 1000 optimism estimates are discarded. The DeLong test was used to perform pairwise comparison between the AUC-ROCs (to demonstrate that the AUC-ROCs of two models are statistically different).

The Youden Index was used to determine the cut-off point on the predicted probabilities that results in the best trade-off between sensitivity and specificity. Based on this cut-off point sensitivity, specificity, positive predictive value, and the negative predictive values were calculated for all models. We used the R statistical environment (version 3.5.3) with R studio using the following packages: pROC, Hmisc, rms, and Resource Selection. This study is reported in accordance to the TRIPOD reporting statement.

## Results

Table 2 shows the baseline characteristics of the included patients. A total of 3604 patients were included during the study period and 2330 patients remained after applying the exclusion criteria. The mean age of the included patients was 61 ± 18 years ranging from 18 to 65. Of the included patients, 53% were male. About 19% of the patients died in hospital.

**Table 2:** Baseline characteristics of study population.

Characteristics	Deceased (*N=*426)	Alive (*N=*1779)	*P-*Value
Age (year)	67.89 ± 15.88	60.38 ± 18.77	<0.001 ^a^
Gender (Male)	232 (54.4%)	944 (53%)	0.62 ^b^
Transfer by EMS	244(57.3%)	775(43.5%)	<0.001^b^
*Clinical parameters*			
Temperature (^0^C)	37.22 ± 0.83	37.26 ± 0.80	0.32 ^a^
Systolic blood pressure (mmHg)	120.62±29.35	128.2±26.55	<0.001 ^a^
Diastolic blood pressure (mmHg)	74.58±18.13	78.18±16.25	<0.001 ^a^
MAP (mmHg)	89.92 ± 20.84	94.85 ± 18.43	<0.001 ^a^
Pulse Rate (beats/min)	100.18 ± 21.66	93.09 ± 19.53	<0.001 ^a^
Respiratory Rate (per min)	22.08 ± 7.22	19.82 ± 5.24	<0.001 ^a^
Peripheral oxygen saturation (%)	90.45±6.93	94.72±5.14	<0.001 ^a^
Abnormal ECG	48(11.26%)	65(3.6%)	<0.001^b^
Ventilation support	93 (21.8%)	30 (1.68%)	<0.001^b^
Glasgow Coma Scale (GCS)	13.51 ± 2.15	14.64 ± 1.00	<0.001 ^a^
Coma without intoxication or overdose	23 (5.3%)	10 (0.56%)	<0.001^b^
Altered mental status without coma	145 (34.03%)	177 (9.94%)	<0.001^b^
AVPU			
-Alert	258 (60.5%)	1592(89%)	<0.001^c^
-Voice Responsive	119 (27.9%)	160 (8.9%)	
-Pain Responsive	26 (6.1%)	17 (1%)	
-Unconscious	23 (5.39%)	10 (0.5%)	
Unable to stand unaided	59 (13.84%)	127 (7.13%)	<0.001^b^
Diabetes	114 (26.7%)	444 (24.9%)	0.457 ^b^
New stroke on presentation	16 (3.75%)	23 (1.29 %)	<0.001^b^
Breathless on presentation	5 (1.17%)	15 (0.84%)	0.567 ^b^
*Laboratory parameters*			
Urea (mg/dL)	113.18 ± 90.99	66.54 ± 59.68	<0.001 ^a^
Creatinine (mg/dL)	2.50 ± 2.53	1.94 ± 2.34	<0.001 ^a^
Sodium (mEq/L)	136.16 ± 8.08	136.59 ± 6.30	0.3 ^a^
Potassium (mEq/L)	4.58 ± 1.17	4.29 ± 0.88	<0.001 ^a^
Albumin (gr/dL)	3.27 ± 0.67	3.61 ± 0.55	<0.001 ^a^
White Blood Cell (10^9^/L)	14.05 ± 13.18	11.38 ± 13.92	<0.001 ^a^
Hemoglobin (gr/dL)	11.76±5.61	12.11±11.73	0.558
Platelet (10^9^/L)	206.45 ± 143.14	226.93 ± 130.44	0.01 ^a^
*ED Risk scores*			
SCS	6.71±3.53	4.08±3.02	<0.001 ^a^
WPS	4.52±2.66	2.44±2.07	<0.001 ^a^
RASP	2.64±2.15	1.47±1.67	<0.001 ^a^
REMS	7.79±3.48	5.48±3.37	<0.001 ^a^
MEWS	3.53±2.65	1.84±2.10	<0.001 ^a^
RLD	145.73±91.7	105.53±60.57	<0.001 ^a^

Values are presented as mean ± SD or N (%)

*Abbreviations*: *ESI* Emergency Severity Index, *FiO*_*2*_ Fraction of inspired oxygen, *PCO*_*2*_ Partial pressure of carbon dioxide, *HCO*_*3*_ Bicarbonate, *MAP* Mean arterial pressure, *GCS* Glasgow Coma Scale, *EMS* Emergency medical services, *SCS* Simple Clinical Score, *WPS* Worthing Physiological Score, *RAPS* Rapid Acute Physiology Score, *REMS* Rapid Emergency Medicine Score, *MEWS* Modified Early Warning Score, *RLD* Routine Laboratory Data

^a^ Analysis by independent-samples t test. ^b^ Analysis by Fisher's exact test. ^c^ Analysis by Chi-square test

Significant differences were observed between the survivors and non-survivors in terms of almost all vital signs and laboratory parameters in addition to ED risk scores, abnormal ECG, and recent stroke events. However, gender, temperature, diabetes, ventilation support, sodium, and hemoglobin levels were not significantly different between the groups.

**Fig. 1:**
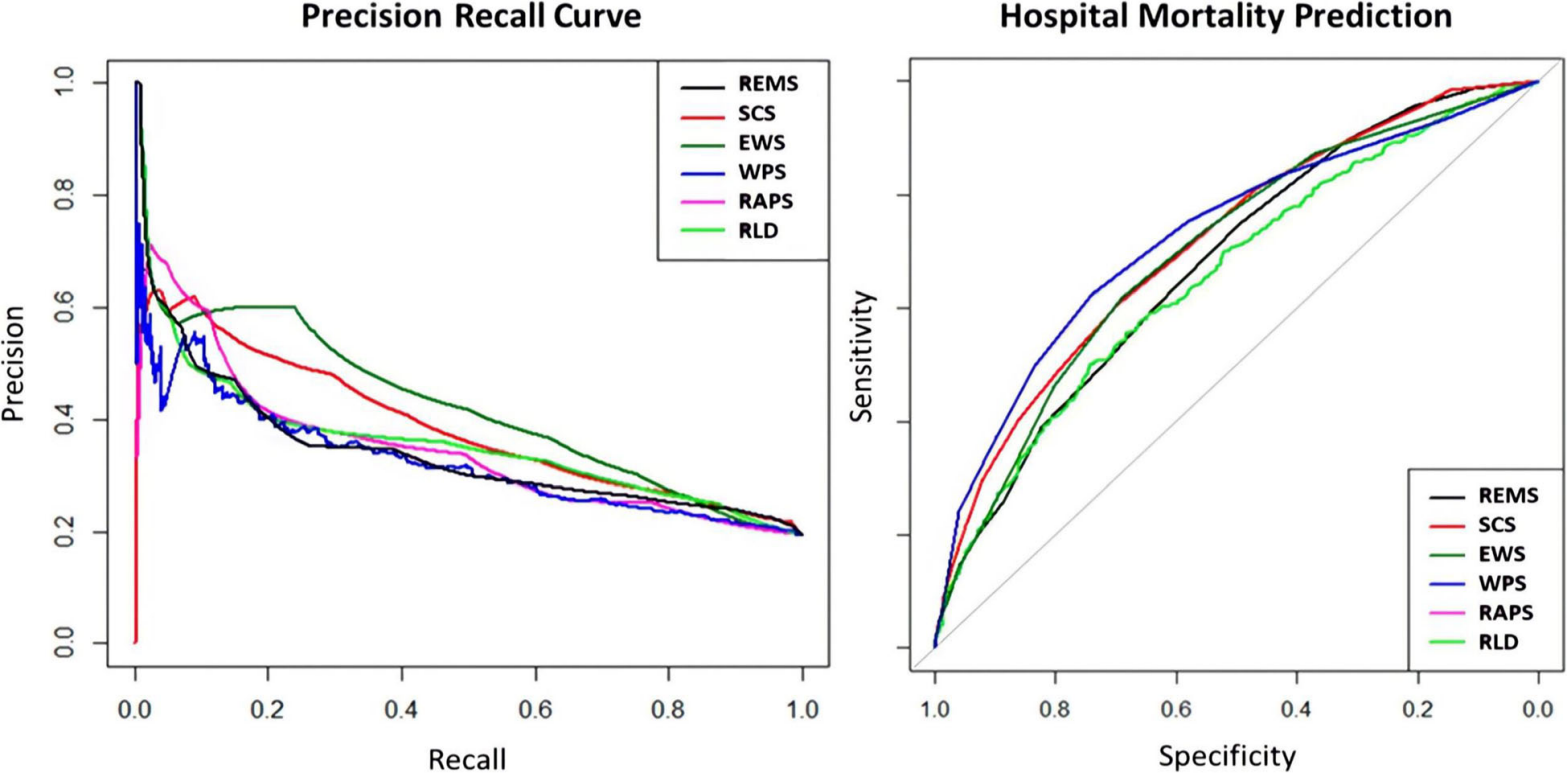
Left: The area under the Precision-Recall (PR) curve represents how a model balances the sensitivity and the positive predictive value. The y-axis represents the precision (positive predictive value in medical terms) and the x-axis represents recall (sensitivity). The AUCPR for SCS, WPS, RAPS, REMS, EWS, and RLD are 0.39, 0.42, 0.35, 0.34, 0.36, and 0.33 respectively. Right: The receiver operating characteristic (ROC) curves graphically represent sensitivity on the y-axis, and 1 - specificity on the x-axis. The area under the curve (AUC) gauges the discriminatory ability of a model. This area was: 0.714 for SCS, 0.727 for WPS, 0.661 for RAPS, REMS 0.678 for REMS, 0.699 for EWS and 0.657 for RLD in the ED.

Table 3 specifies the final models of all scoring systems in terms of their linear predictors. The table also shows the optimism-corrected performance measures. The WPS, SCS and MEWS have the highest optimism-corrected discrimination ability compared to the other models (see also Fig. 1). Pairwise comparisons of the AUC-ROCs are presented in Table 4. The SCS, WPS, and MEWS had a higher discrimination for prediction of in-hospital mortality among critically ill patients who are presented to the ED (AUC-ROC of 0.71, 0.73, and 0.70, respectively). The RAPS, REMS, and RLD models showed lower discrimination (AUC-ROCs < 0.68). In terms of discrimination power, the WPS model was significantly better than its counterparts except for SCS (*P-*value = 0.242). Moreover, the WPS, SCS, and MEWS had higher AUC-PR (0.42, 0.39, 0.36 respectively) which shows their ability to better balance sensitivity and the positive predictive value.

**Table 3:** Intercept and slope of the linear predictor of the logistic regression for all models to predict in-hospital mortality in ED; the optimism-corrected performance measures; and various threshold-based metrics (the threshold is itself based on the Youden index)

Models	Intercept (***β***_**0**_)	Slope (***β***_**1**_)	AUC-ROC (95% CI)	AUC-PR (95%CI)	BS	BS 95% CI	Threshold^a^	SE	SP	PPV	NPV	Accuracy
SCS	-2.6914	0.238	0.71(0.688, 0.742)	0.39 (0.37-0.41)	0.146	(0.136,0.154)	5.5	0.607	0.697	0.3249	0.881	0.680
WPS	-2.6456	0.3583	0.73(0.700, 0.757)	0.42 (0.40-0.44)	0.173	(0.164,0.182)	3.5	0.624	0.7403	0.3653	0.8916	0.717
RAPS	-2.0675	0.318	0.66(0.635, 0.686)	0.35 (0.33-0.37)	0.091	(0.079,0.098)	1.6	0.495	0.7667	0.3370	0.8638	0.714
REMS	-2.692	0.1913	0.68(0.657, 0.705)	0.34 (0.32-0.36)	0.10	(0.095,0.112)	5.5	0.748	0.4957	0.2623	0.8918	0.544
MEWS	-2.1653	0.2833	0.70(0.678, 0.726)	0.36 (0.34-0.39)	0.16	(0.105,0.123)	2.5	0.617	0.693	0.3250	0.8832	0.678
RLD	-2.3523	0.0076	0.66(0.632, 0.689)	0.33 (0.31,0.35)	0.07	(0.060,0.082)	127	0.5	0.7431	0.3179	0.8612	0.6961

*Abbreviations*: *AUC-ROC* Area Under the receiver operating characteristic Curve, *AUC-PR* The area under the precision-recall curve, *CI* Confidence Interval, *BS* Brier Score, *PPV* Positive Predictive Value, *SE* sensitivity, *SP* specificity, *NPV* Negative Predictive Value, *SCS* Simple Clinical Score, *WPS* Worthing physiological score, *RAPS* Rapid Acute Physiology Score, *REMS* Rapid Emergency Medicine score, *MEWS* Modified Early Warning Score, *RLD* Routine Laboratory Data, *ED* Emergency Department

^a^ This threshold is calculated based on the Youden index

**Table 4:** Pairwise comparison of AUCs by using the DeLong test for each pair of models.

DeLong	WPS	RAPS	REMS	MEWS	RLD
SCS	(-0.0087,0.0331)	(-0.0820,-0.0233)	(-0.0549,-0.0148 )	(-0.0408,0.0070)	(-0.0936,-0.0236)
	*p-*value = 0.242	*p-*value = 0.0006	*p-*value = 0.0007	*p-*value = 0.2008	*p-*value = 0.0021
WPS		(-0.0909,-0.0392)	(-0.0744,-0.0224)	(-0.0454,-0.0115)	(-0.1080,-0.0325)
		*p-*value = 1.901e-06	*p-*value = 0.0003	*p-*value = 0.0006	*p-*value = 0.0004
RAPS			(-0.0071,0.0408)	(0.0163,0.0622)	(-0.0469,0.0372)
			*p-*value = 0.1584	*p-*value = 0.0014	*p-*value = 0.8292
REMS				(-0.0050,0.0425)	(-0.0586,0.0136)
				*p-*value = 0.0971	*p-*value = 0.2623
MEWS					(-0.0828,-0.0015)
					*p-*value = 0.0366

*Abbreviations*: *SCS* Simple Clinical Score, *WPS* Worthing physiological score, *RAPS* Rapid Acute Physiology Score, *REMS* Rapid Emergency Medicine score, *MEWS* Modified Early Warning Score, *RLD* Routine Laboratory Data

**Fig. 2:**
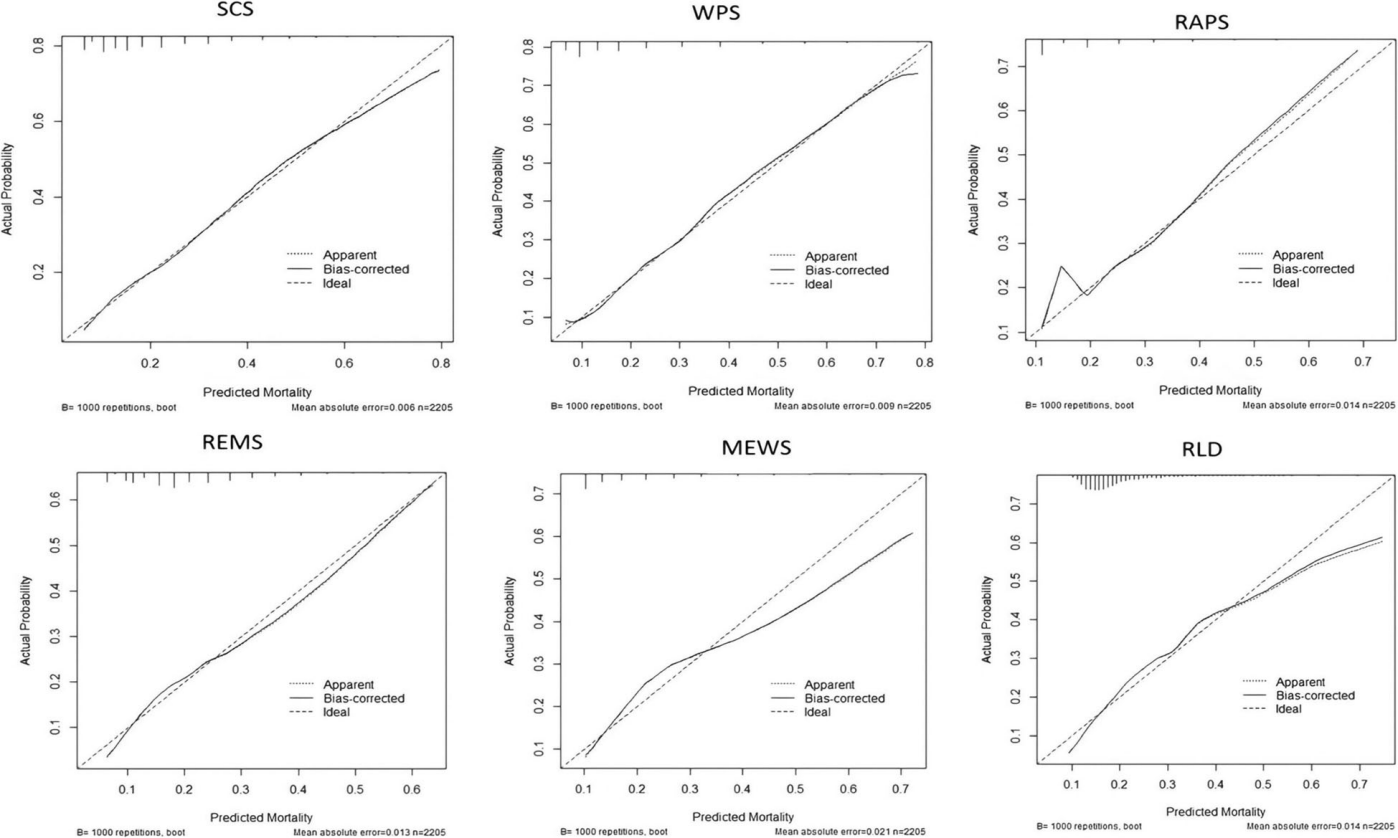
Calibration plots of the six models. A calibration plot is a measure of goodness-of-fit as a graphical presentation of the actual mortality probability versus the predicted mortality probability. The calibration plots of SCS, WPS and REMS do not deviate much from the diagonal line, which represents perfect calibration

Figure 2 shows the calibration plots of the six models. It is apparent that the degree of correspondence between the predicted and observed probabilities vary markedly between the models and that the calibration of the SCS, WPS, and REMS show good correspondence.

## Discussion

Severe overcrowding and shortage of resources (esp. personnel and medical equipment) have remained a concerning issues in any ED setting. The problem seems to be more prominent in developing countries. Accurate assessment and identification of the patients who are in high need of critical care is the most challenging task. Employing scoring systems has been suggested to achieve optimal use of limited resources. Furthermore, several previous studies have suggested the advantage of using scoring systems in improving patient turnover, resource allocation, and benchmarking [3].

### Main findings

We performed a comparison of six scoring systems, in terms of their predictive performance. We found that the WPS had superior discrimination than the other models except for SCS (*p =* 0.242). The WPS had higher AUC-PR as well, which means this model provides a better balance between the positive predictive value and sensitivity across the graph. With respect to the overall performance of the accuracy of the predicted probabilities as measured by the Brier score, the RLD had the lowest value while the WPS has the highest. The Brier score indicates the errors between the predictions and actual outcomes. In general, the WPS and RAPS models had the highest accuracy compared with the other models. RAPS, RLD, and WPS showed the highest specificity values, and REMS showed the highest sensitivity value. However, in comparison with other models, REMS had the lowest specificity value. A model with high sensitivity but low specificity could be suitable for preliminary screening. On the other hand, a model with high specificity but relatively low sensitivity, could be more suitable for assigning individuals to a high-risk intervention. In the latter case, it is appropriate for assigning patients with high priority for CPR or the ICU where it is fully equipped with high-tech devices for resuscitation. Such a model is useful for individuals with high risk. The expected benefit is then proportional to the prevalence.

The WPS and SCS and REMS models showed good agreement between observed and predicted probabilities of in-hospital mortality during the entire range of predicted probabilities. The other models showed worse deviation from the diagonal line indicating their tendency to underestimate or overestimate the in-hospital mortality rate (Fig. 2). MEWS and RLD overestimate the mortality rate for the probabilities larger than 0.40. In contrast, RAPS underestimate the mortality rate in that range.

Furthermore, an NPV value greater than 0.86 for all models indicate that these models predict alive patients better than the *deceased* ones. This implies that in this population, more than 86% of the patients predicted to survive, have indeed survived. Since the PPV and NPV are ratios that includes both alive and deceased subjects, the predictive values are affected by the prevalence of the deceased cases and can differ between settings. The lower the prevalence of the deceased cases, the higher its NPV. On the other hand, the higher the prevalence of the deceased cases, the higher the PPV.

A potential important reason for the relatively low AUROC compared to its value for the original models, is that the original models are based on western populations and we now apply them on an Asian population. In addition, there might be differences in the type of equipment, the care methods, and treatment policies.

Generally, in clinical applications practicality and clinical sensibility are important, necessitating the use of a clear and interpretable clinical decision method. The need for a concise decision method could be much more pressing in the ED, where physicians often have no time to review patients due to the stressful environment. Models with more variables and complex non-linear functions of continuous predictors have the potential to perform better and provide more accurate predictions in general. Some researchers contend that reducing the complexity of models by categorizing continuous predictors or omitting predictors from a model is inappropriate since these techniques may have a negative impact on the model’s predictive performance. The aim of developing a prediction model is to provide a reliable model that can be transportable and adopted in clinical practice; therefore, it is important to settle on a relatively parsimonious model that does not forfeit significant predictive performance. Interestingly, our present study indicated that models with fewer variables such as the WPS and the MEWS performed similarly to or even better than models with more variables such as the SCS and the RLD.

### Comparison to similar studies

Emergency models have been previously evaluated in different EDs around the world. However, to the best of our knowledge, this is the most comprehensive study comparing the predictive performance of the models based on six scoring systems (SCS, WPS, RAPS, REMS, MEWS, and RLD) to predict in-hospital mortality in a large sample of patients admitted to the emergency department. Table 5 lists and compares various studies performed in the ED settings.

**Table 5:** Published evaluation studies of the SCS, WPS, RAPS, REMS, MEWS and RLD models in the emergency department

Study	Year	country	Sample Size (N)	Male Gender (%)	Age	Mortality Rate (%)	DX.	Cut-off point	Prediction Score	AUC-ROC (95%CI) or AUC-ROC ± SE	Calibration(H-L) or Intercept, slope	NO. of Center (N)
[18]	2015	Vietnam	1746	45 %	68 (55, 80)	9.9 %	Case-mix	NA	WPS =2 (2,4)	WPS=0.80 (0.76 to 0.83)	Adequate	Single Center
									REMS= 6 (5,8)		WPS; (*P* >0.05)	
										REMS=0.712 (0.67 to 0.76)	REMS; (*P>*0.05)	
[3]	2019	Turkey	250	57.6 %	57.6 ± 20.82	21.6 %	Case-mix	MEWS ≥4	MEWS=3.43± 2.34	MEWS=0.71 (0.711 ± 0.039)	NA	Single Center
								WPS	WPS =4.56 ±			
										WPS = 0.7 7 (0.769 ±0.034)		
								≥5 REMS ≥6	3.13 REMS=7.48± 4.74			
										REMS=0.70 (0.703 ± 0.036)		
[19]	2014	Turkey	2000	52 %	61.41±18.92	7.7 %	Case-mix	NA	REMS=5 (0,17)	REMS=0.71 (0.67 to 0.72	NA	Multi Center=3
									MEWS=1 (0,9)	MEWS=0.63 (0.61to 0.65)		
[20]	2014	USA	3680	75 %	43.7 ±21	5.2 %	Trauma	NA	REMS=3.4 ± 3.2	REMS=0.91 (0.91±0.02)	NA	Single Center
[2]	2014	USA	227	51%	65 ± 17	25%	Case-mix	NA	NA	REMS=0.70 (0.62, 0.78)	Adequate	Single Center
										MEWS=0.70 (0.62,0.77)	REMS; (*P* >0.05)	
											MEWS; (*P>*0.05)	
[21]	2013	Germany	151	54.3 %	68.3 ± 18	NA	Sepsis	MEWS ≥5	MEWS=3.32 ±NA	MEWS=0.641 (0.552to 0.730)	NA	Single Center
[22]	2015	Germany	5730	55.5 %	61.2±17.7	21 %	Patients with sinus rhythm	NA	MEWS=for	MEWS=0.71 (0.67–0.75)	NA	Single Center
									Survival=3.5 ± 1.7			
									Unsurvival=2.3 ±1.4			
[23]	2018	China	4857	47.9 %	44.5±18.3	4.38 %	Case-mix	REMS≥ 8	NA	REMS=0.88 (0.86 to 0.90)	Inadequate for all scores (*P<*0.001)	Single Center
								RAPS≥7		RAPS = 0.72 (0.69 to 0.77)		
								MEWS≥6		MEWS= 0.65 ( 0.69 to 0.78)		
[24]	2014	China	234	58.5%	65.8 ±18.1	NA	Case-mix	NA	NA	MEWS= 0.73 (0.67 to 0.79)	NA	single Center
										REMS=0.70 (0.64 to 0.76)		
										SCS=0.70 ( 0.64 to 0.76)		
[25]	2017	Denmark	5784	50.4%	67 (49–78)	24.6%	Case-mix	NA	NA	WPS= 0.8 (0.73 to 0.86)	Adequate	Single Center
										RAPS=0.61 (0.54 to 0.69)	WPS; (*P>*0.05)	
										REMS=0.77 (0.72 to 0.83)	RAPS; (*P=*0.05)	
											REMS; (*P>*0.05)	
[26]	2017	Taiwan	114	67.54%	56.33±16.12	28.58%	Splenic abscess	MEWS≥6	NA	MEWS=0.76	NA	Multi Center=4
								RAPS≥4		RAPS=0.68		
								REMS≥7		REMS=0.67		
[27]	2017	Taiwan	66	54.55%	69.23 ± 16.64	57%	HPVG	MEWS≥6	MEWS= 6.94 ± 3.46	MEWS= 0.8562	Adequate	Multi Center=2
								RAPS≥4	RAPS= 5.97 ± 4.15	RAPS= 0.8769 (NA)	MEWS;(*P>*0.05)	
								REMS≥11	REMS= 1.09 ± 5.23	REMS= 0.9286 (NA)	RAPS;(*P>*0.05)	
											REMS;(*P>*0.05)	
[28]	2011	Israel	1072	52.2%	74.7 ± 16.1	21.9 %	Sepsis	NA	MEWS= 3.48±2.24	MEWS= 0.69 (0.65 to 0.70)	Adequate	Single Center
									SCS^*^= 12.09 ±3.62	SCS= 0.77 (0.74 to 0.80)	MEWS;(*P>*0.05)	
									REMS= 9.17±4.06	REMS= 0.77 (0.73 to 0.80)	SCS; (*P>*0.05)	
											REMS; (*P>*0.05)	
[29]	2010	Ireland	270	50%	66.56 ± 18.2	NA	Case-mix	NA	NA	SCS=0.94 ( NA)	NA	Single center
[30]	2017	South Korea	6905	62.2 %	57.42± 18.51	3 %	Trauma	REMS= 7	REMS= 4.48± 3.03	REMS = 0.90	NA	Single center
[16]	2016	UK	24696	29 %	63.1 ± 21.1	4.69%	Case -mix	Na	NA	RLD =0.83 (0.823 to 0.842)	RLD; (*P>*0.05)	Single center
										RLD or BHOM	RLD=BHOM	
[31]	2017	Iran	2148	75.56%	39.50±17.27	5.73 %	Trauma	REMS ≥ 3	NA	REMS= 0.93 (0.92 to 0.95)	Adequate	Multi Center= 4
								RAPS ≥ 2		RAPS= 0.899 (0.86 to 0.93)	REMS (0.001,0.98)	
											RAPS=(0.003,0.96)	
[32]	2016	Iran	735	75.37	41.08±18.46	6.53%	Trauma	RAPS≥5 WPS≥2	NA	RAPS=0.93 (0.88-0.98)	NA	Single center
										WPS= 0.97 (0.96-0.98)		
*Present study*	2018	Iran	2,330	53%	61 ± 18	19 %	Case-mix	SCS ≥5.5	SCS= 4.59±3.29	SCS= 0.714 (0.688 to 0.742)	Adequate	Single center
								WPS ≥3.5	WPS= 2.84±2.35		Graphically for WPS	
								RAPS ≥1.6	RAPS= 1.70±1.84	WPS=0.7272 (0.70 to 0.757)		
								REMS ≥ 5.5	REMS= 5.92±3.51		SCS	
								MEWS ≥2.5	EWS= 2.17±2.31	RAPS=0.661 (0.635 to 0.69)	Inadequate for others	
								RLD ≥ 127	RLD=113.29±69.5			
										REMS=0.678 (0.657 to0.71)		
										MEWS=0.698 (0.68 to 0.73)		
										RLD=0.656 (0.632 to 0.689)		

As shown in Table 5, the lowest and highest mortality rates in similar studies were 3 and 57%, respectively. Moreover, the largest sample size belongs to the study on RLD (BHOM) with 24,696 participants with a 4.69% mortality rate. The median sample size of the similar studies was 1746 (IQR: 234–4857, min-max: 66–24,696). In 14 out of 17 studies, males formed the majority of participants.

The majority of the studies were single-centered studies. Some included patients with specific diseases such as Hepatic portal venous gas (HPVG), splenic abscess, and trauma from different centers [20, 25, 27, 31]. Findings of three studies showed that WPS was superior to REMS [3, 27, 33] which is consistent with the results of the current study. Moreover, Mirbaha et al. reported similar predictive performance for the WPS and a short version of REMS (RAPS) [30].

The Rapid Acute Physiology Score was developed in a different setting and patient population than the rest of the scoring systems. This system takes those elements of APACHE-II that can be obtained reliably on all patients in a hospital emergency department. It is still meaningful to compare this model to the other scoring systems, as has been done for example in [23–25].

As shown in Table 5, REMS is the most commonly evaluated model in the previously published studies. Of these studies, the REMS has excellent discrimination among patients who suffer from HPVG and trauma (AUC-ROC > = 0.90) while among most of studies inspecting the REMS on heterogeneous patients, the discrimination ability was in the fair range (AUC-ROC between 0.7 and 0.8).

Several studies have reported that REMS was superior to MEWS (2011 to 2019 in Israel, Taiwan, China, and Turkey) [20, 23, 24, 31], which is in contrast to the results of the current study and other evidence presented in Table 5 [3, 22, 27]. Consistent with our findings, researches from the United States and Turkey indicate that the performance of these two models is similar to each other [2, 3]. As demonstrated in Table 5, MEWS was associated with fair discrimination in five studies, besides the current study [2, 3, 21, 22, 27]. However, in contrast four studies reported poor AUC-ROCs [20, 23, 24, 29]. There weren’t any significant differences between the SCS and MEWS in terms of discriminatory ability which is in contrast to studies performed in China and Israel [24, 28].

In respect of calibration as presented in Table 5, WPS and REMS had fair calibration in four studies [2, 18, 25, 27, 31]. The RLD model was also associated with fair calibration in one study [16]. In contrast, one study reported inadequate calibration for REMS, RAPS, and MEWS [23]. It should be noted that the majority of studies used the Hosmer-Lemeshow goodness-of-fit test to evaluate the calibration. However, this test has some disadvantages, including sensitivity to the sample size (the larger the sample size the more the test tends to show significant deviations from the ideal calibration). Moreover, the test provides no information about the range of predicted probabilities where the model overestimates or underestimates the outcome variable [34].

This study has also limitations. First, we conducted a single center study which limits the generalizability of the results. However, this center was considered as the largest referral emergency department in the northeast of the country and included a wide spectrum of diseases. Second, exclusion of the patients who were referred to the special EDs (e.g. trauma, obstetrics, and etc.) results in the inapplicability of the models for these groups of patients.

## Conclusions

In comparison to other models, the SCS and WPS revealed more successful discrimination in prediction in-hospital mortality. Moreover, SCS and WPS calibration plots showed good agreement between the predicted and observed mortality probabilities. There was no significant difference between the AUC-ROC of the SCS and WPS models. All models may benefit from recalibration on the external datasets and further validation studies are needed before warranting routine clinical use. Aside from the potential benefit from recalibration on the external datasets, and further validation studies, future studies should also attempt to develop more sensitive scoring systems before warranting routine clinical use.

## Data Availability

The datasets generated and/or analyzed during the current study are not publicly available due [REASON WHY DATA ARE NOT PUBLIC] but are available from the corresponding author on reasonable request.
